# Peripheral blood transcriptomic analysis identifies potential inflammation and immune signatures for central retinal artery occlusion

**DOI:** 10.1038/s41598-024-57052-8

**Published:** 2024-03-28

**Authors:** Jiaqing Feng, Ying Li, Chuansen Wang, Yuedan Wang, Yuwei Wan, Mengxue Zheng, Ting Chen, Xuan Xiao

**Affiliations:** 1 Department of Ophthalmology, Renmin Hospital of Wuhan University, No. 238 Jie Fang Road, Wuhan, 430060 Hubei China; 2 Department of Clinical Laboratory, Institute of Translational Medicine, Renmin Hospital of Wuhan University, Wuhan, China

**Keywords:** Central retinal artery occlusion, Transcriptomics in CRAO, Biomarkers, Ribosomal proteins, Data processing, Gene regulatory networks, Biomarkers, Retinal diseases

## Abstract

Central retinal artery occlusion (CRAO) is an acute retinal ischaemic disease, but early diagnosis is challenging due to a lack of biomarkers. Blood samples were collected from CRAO patients and cataract patients. Gene expression profiles were distinct between arterial/venous CRAO blood (A–V group) and venous CRAO/control blood (V–C group) samples. Differentially expressed genes (DEGs) were subjected to GO and KEGG enrichment analyses. Hub genes were identified by Cytoscape and used to predict gene interactions via GeneMANIA. Immune cell infiltration was analysed by CIBERSORT. More than 1400 DEGs were identified in the A–V group and 112 DEGs in the V–C group compared to controls. The DEGs in both groups were enriched in the ribosome pathway, and those in the V–C group were also enriched in antigen processing/MHC pathways. Network analysis identified ribosomal proteins (*RPS2* and *RPS5*) as the core genes of the A–V group and MHC genes (*HLA-F*) as the core genes of the V–C group. Coexpression networks showed ribosomal involvement in both groups, with additional immune responses in the V–C group. Immune cell analysis indicated increased numbers of neutrophils and T cells. Ribosomal and MHC-related genes were identified as potential CRAO biomarkers, providing research directions for prevention, diagnosis, treatment and prognosis.

## Introduction

Central retinal artery occlusion (CRAO) is an ophthalmic emergency characterized by acute, painless, monocular vision loss^1^. Thrombotic or embolic occlusion of the retinal artery can irreversibly damage the retina within 90–240 min^2^. Patients with CRAO have an increased risk for future cardiovascular events, such as stroke and myocardial infarction, as well as increased all-cause mortality^3^. Given that spontaneous recanalization of the central retinal artery rarely occurs, rapid treatment is critical^4^. However, current treatments demonstrate suboptimal efficacy. Even with timely treatment, further eye tissue damage persists under reperfusion, leading to unsatisfactory visual prognoses^5^. Further research into the pathogenesis of CRAO is warranted to explore more effective therapies and improve patient outcomes.

In addition to vascular factors, other related molecular mechanisms contribute to CRAO progression and outcomes, including key pathways such as the *AKT1*, *JAK-STAT*, *MAPK*, and *HIF1A* pathways^6–8^. Retinal ischaemia‒reperfusion leads to early stabilization of hypoxia-inducible factors and upregulation of hypoxia-responsive genes^9^. Transient increases in *CCL2* and decreases in *IL-27* occur early on. This is followed by late activation of astrocytes and Müller cells, as well as progressive inner retinal thinning. Molecular analysis revealed early decreased expression of the retinal ganglion cell marker *Thy1* and late decreased expression of the amacrine cell marker *Sncg*, indicating ischaemic damage and loss of these neuronal cells over time^9^. Recent studies have also demonstrated that transcriptome analysis of peripheral blood monocytes can reveal differences in immune status between individuals and can assist in the diagnosis and prediction of eye diseases^10,11^. However, research into the underlying mechanisms of CRAO is limited by the scarcity of human retinal samples. Many studies have relied on animal models or circulating blood, but transcriptomic approaches to identify CRAO-related genes in human samples are lacking.

To address this gap in knowledge, this study aimed to elucidate CRAO mechanisms using RNA-seq transcriptomic profiling of accessible samples. Through transcriptome technology, key transcription factors and signalling pathways that play crucial roles in the differentiation of retinal cells have been identified^12^. Additionally, transcriptomic analysis can identify hub genes and key pathways with potential clinical utility^13–16^ and provide targets for disease treatment^17^. Blood samples from CRAO patients and control individuals were subjected to RNA sequencing and bioinformatic analysis to reveal differentially expressed genes (DEGs) and dysregulated pathways, which may contribute to retinal damage in patients with CRAO. Achieving these aims will provide insights into the pathogenesis of CRAO and establish a foundation for developing targeted therapies.

## Material and methods

### Patients recruitment and sample collection

We collected blood samples from 16 CRAO patients and 9 cataract controls at Wuhan People's Hospital in China. CRAO patients were included if they had clinical and angiographic diagnosis of CRAO less than 72 h after symptom onset, best-corrected visual acuity between counting fingers and 20/50, and availability of peripheral vein or arterial embolus site samples before thrombolysis. Patients were excluded if they had glaucoma, retinal or disc neovascularization, any previous CRAO treatment, non-CRAO vascular retinopathy, intraocular surgery in the prior 3 months, stroke, myocardial infarction, hemi-CRAO, macula-sparing CRAO, or other severe ocular diseases. The CRAO cohort aligned with published diagnostic guidelines^18^ including sudden vision loss, positive RAPD, retinal ischemic edema, and delayed arterial filling on angiography. Cataract controls had surgery during the same period. Additional exclusions for both groups were prior retinal artery occlusion, coronary artery disease, malignancy, severe renal insufficiency (eGFR < 30 mL/min), liver disease, stroke, or lung disease. All arterial CRAO blood samples were collected during central retinal artery interventional surgery. The ethics committee of Wuhan People's Hospital approved this study (protocol code, WDRY2022-K278 and approval date, 30 November 2022), which adhered to the 1995 Declaration of Helsinki guidelines. All participants provided informed consent.

### Peripheral blood mononuclear cells (PBMCs) isolation and RNA sequencing library construction

Fresh venous anticoagulant blood samples (VBS) or arterial anticoagulant blood samples (ABS) for CRAO were obtained and blood mononuclear cells were isolated using a Ficoll separation gradient. Total RNA was extracted using Trizol reagent. RNA quality control was performed to assess purity, concentration, integrity and quantity. For RNA sequencing, 3 μg of total RNA was used as input. mRNA was purified and fragmented. cDNA was synthesized and amplified to generate sequencing libraries. Libraries were quantified and sequenced on the DNBSEQ-T7 platform using 150 bp paired-end reads. The serum samples used in this study were collected by EDTA-coated procoagulant tubes and centrifugated before frozen at − 80 °C.

Following final library quality control on the Agilent Bioanalyzer to confirm the expected size distributions, libraries were pooled and sequenced on the Illumina HiSeq3000 platform in a 150-bp paired-end read run format. Raw RNA sequencing (RNA-seq) reads were preprocessed and determined using featureCounts function in SubReads package v1.5.3 with default parameter (Parameter setting is -t exon -g gene id -s 0 -*p* > {log} 2 > &1).

### Bioinformatic analysis of microarray result

#### Differential expression analysis and identification of RNAs

Quantile normalization and subsequent data processing were performed using the 'limma' package in R software (version 4.2.3)^19^. Principal Component Analysis (PCA) was conducted to investigate group differences based on the variables of interest. For 3D PCA analysis and plot generation, the 'stats' and 'ggplot2' packages in R were utilized. Volcano plots were employed to identify significant differentially expressed genes (DEGs) between the Artery group and the Vein group, as well as between the Vein group and the control group. Genes with |log2 fold change|≥ 2 and adjusted *p*-value < 0.05 were considered statistically significant in both the A–V and V–C groups. Additionally, hierarchical clustering was performed to reveal distinguishable RNA expression patterns among the samples, and the 'pheatmap' package was used to visualize the expression patterns of the 50 most significantly differentially expressed genes (DGEs) in a heatmap.

#### GO and KEGG enrichment analysis

The Gene Ontology (GO; http://geneontology.org) is a structured and computable knowledge resource that aids in identifying the molecular function (MF), biological process (BP), and cellular component (CC) attributes of genes^20^. It is particularly useful for analyzing high-throughput genome or transcriptome data. The Kyoto Encyclopedia of Genes and Genomes (KEGG; https://www.kegg.jp/) is a meticulously curated resource that enables systematic analysis of gene functions and associations by linking gene sets to corresponding pathways^21^. GO annotation and KEGG pathway enrichment analyses were performed using the 'clusterProfiler' package in R (version 4.2.3)^22^. The enrichment functions and pathways of the DEGs were determined by applying a threshold of adjusted *p* < 0.05 and a count of enriched genes greater than five.

#### Gene set enrichment analysis

The study employed the Gene Set Enrichment Analysis (GSEA) program (version 4.3.2) with default settings to investigate significant functional and pathway differences between the Artery groups and Vein groups, as well as between the Vein groups and the control group. The objective was to elucidate the underlying molecular distinctions^23^. Pathways enriched for each phenotype were obtained from the h subset (h.all.v2023.1.Hs.symbols.gmt) set, which served as the preset gene sets for enrichment analysis. Enriched pathways were classified based on criteria such as the adjusted *p*-value (< 0.05), FDR q-value (< 0.25), and normalized enrichment score (|NES|> 1).

#### Building protein–protein interaction networks and identifying hub genes

A protein–protein interaction (PPI) network was constructed to investigate the interaction among DEGs. The Search Tool for the Retrieval of Interacting Genes (STRING) database was utilized to provide a comprehensive score for each PPI relationship pair^24^. Statistically significant interactions were identified by applying a combined score threshold of 0.7. The PPI pairs with an interaction score > 0.7 were extracted and visualized using Cytoscape 3.9.0^25^. To explore the most three key functional modules, the Cytoscape plug-in molecular complex detection (MCODE) was applied to the PPI network. Furthermore, KEGG and GO analyses of the identified modular genes were performed in R and the results were visualized using bar plots.

#### Selection and analysis of hub genes

The CytoHubba plug-in of Cytoscape identified hub genes from the PPI network, and then five algorithms (Radiality, MCC, MNC, Degree and EPC) were used to confirm the final hub genes, which were illustrated in Venn diagram. Ultimately, the above hub genes were submitted to GeneMANIA (http://genemania.org) to construct a co-expression network.

#### Immune cell infiltration analysis

Immune cell infiltration was assessed using the Cibersort algorithm in R software^26^. The mRNA expression matrix (normalized) was used as input to calculate the cell abundance score for 22 cell types. Stacked bar plots were generated by Chiplot (https://www.chiplot.online). Box plots were generated using the 'ggplot2' package, while heatmaps were created using the 'pheatmap' package.

#### Statistical analysis

SPSS (version 27.0; IBM, New York, United States) was used to perform statistical analysis. Continuous data were expressed as mean ± SD and compared by using ANOVA. Categorical variables were presented as counts (frequency) and compared using chi-square test or the Fisher exact test, as appropriate. In some analyses, more than 20% of the cells had expected frequencies less than 5, precluding calculation of *p*-values. A two-sided *p* < 0.05 was considered to statistical significant.

### Ethical approval

The study was conducted according to the guidelines of the Declaration of Helsinki, and approved by the Ethical Committee Board of Renmin Hospital of Wuhan University (protocol code, WDRY2022-K278 and approval date, 30 November 2022).

### Informed consent

As this was a retrospective observational study, patient consent was waived.

## Results

### Clinical characteristics of the recruited patients

Table 1 presents the baseline characteristics of the study participants. A total of 25 patients were included: 16 RAO patients (9 peripheral and 7 arterial) and 9 cataract control individuals. The RAO patients and control individuals were well matched in terms of age (average 63 years), sex (64.00% male), and blood pressure (*p* > 0.05 across groups).

**Table 1:** Comparison baseline demographic data of patients (the values are presented as mean ± SD or as percentages).

	Cataract Control (*n* = 9)	Peripheral Blood Samples (*n* = 9)	Arterial Blood Sample (*n* = 7)	*p* value
Male (%)	6 (66.67)	6 (66.67)	4 (57.14)	0.906
Age (year)	66.78 ± 7.678	61.33 ± 9.22	60.29 ± 7.32	0.238
Systolic blood pressure (mmHg)	140.75 ± 5.91	160.86 ± 23.09	136.50 ± 13.23	0.091
Diastolic blood pressure (mmHg)	82.00 ± 8.79	88.86 ± 13.64	86.00 ± 11.78	0.674
Diabetes mellitus (%)	1 (11.11)	0	1 (14.29)	/
Hypertension (%)	5 (55.56)	1 (11.11)	3 (42.86)	/
Hyperlipidemia (%)	0	0	1 (14.29)	/
Smoking (%)	2 (22.22)	1 (11.11)	0	/
Alcoholic (%)	0	1 (11.11)	0	/

“/” Means more than 20% of the cells had expected frequencies less than 5.

### DEGs between retinal arterial obstruction patients and control individuals

**Figure 1:**
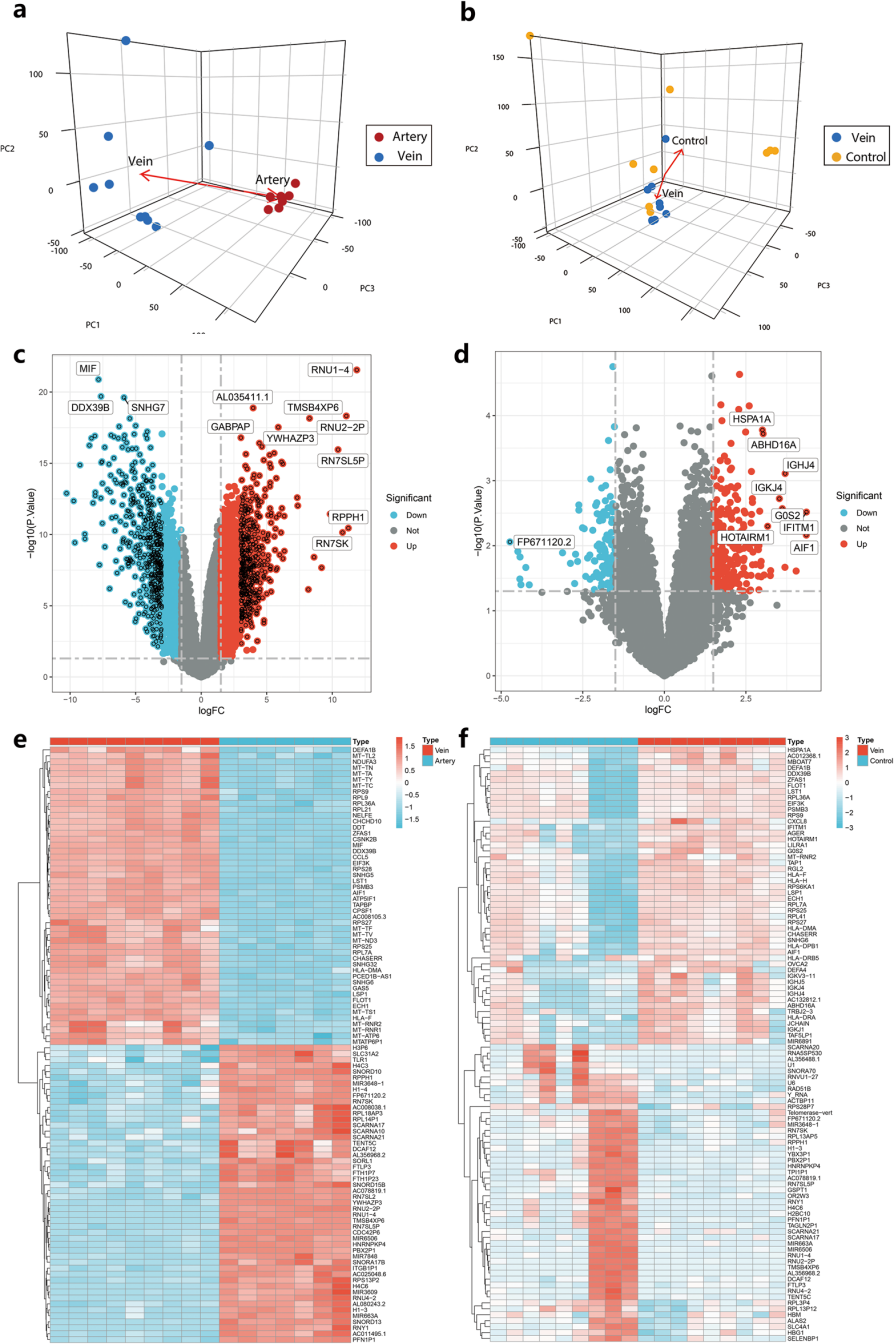
The DEGs between CRAO patients and controls. (**a**) PCA plot of arterial vs venous blood samples. Red dots represent CRAO arterial blood samples. Blue dots represent CRAO venous blood samples. (**b**) PCA plot of venous vs control blood samples. Blue dots represent CRAO venous blood samples. Yellow dots represent cataract control blood samples. (**c**) Volcano plot showing the DEGs of A–V group. (**d**) Volcano plot showing the DEGs of V–C group. (**e**) Heat map of DEGs in A–V group. (**f**) Heat map of DEGs in V–C group.

RNA-seq analyses of ABS, VBS, and control venous blood were conducted to evaluate gene expression changes in PBMCs. Principal component analysis (PCA) revealed a clear separation between CRAO arterial samples and venous samples (A–V group), as well as between CRAO venous samples and control samples (V–C group) (Fig. 1a and b). In the A–V group, 1400 genes were upregulated and 1291 genes were downregulated compared to those in the control group (Fig. 1c and e). In the V–C group, 112 genes were upregulated and 34 genes were downregulated compared to those in the control group (Fig. 1d and f). Functional enrichment analysis provided insights into the biological functions associated with these DEGs.

### Functional enrichment analysis of DEGs

**Figure 2:**
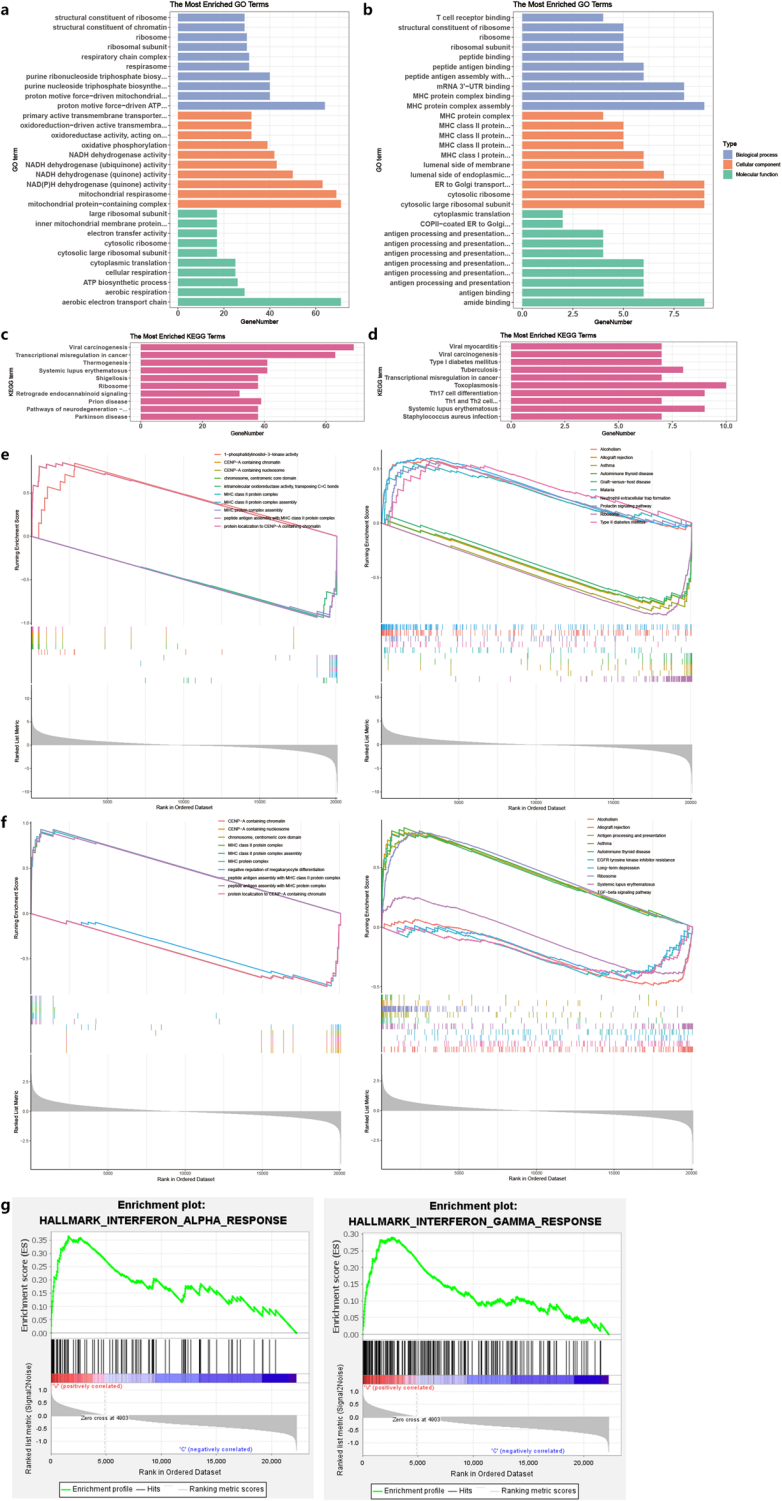
Functional enrichment of DEGs. GO functional analysis of DEGs in A–V group (**a**) and V–C group (**b**). KEGG functional analysis of DEGs in A–V group (**c**) and V–C group (**d**). The gene sets enriched in comparison to ABS **(e)** and VBS (**f**) in the GSEA results. (**g**) GSEA results indicate activation of the antigen-presenting activity pathway in the V–C group (INTERFERON_ALPHA_RESPONSE: *NES* = 2.56 *p-value* < 0.001 *FDR q-value* < 0.001; INTERFERON_GAMMA_RESPONSE: *NES* = 2.23 *p-value* < 0.001 *FDR q-value* < 0.001).

GO and KEGG analyses were performed on the DEGs to determine their biological functions and investigate their roles. DEGs were hierarchically clustered based on GO categories: biological process (BP), molecular function (MF), and cellular component (CC). The top 10 enriched GO and KEGG pathways were identified for each category (Fig. 2a–d). Hierarchical clustering of GO categories revealed enriched pathways in both the A–V and V–C groups, including “ribosomes”, “cytoplasmic translation”, “chromatin remodelling”, “NAD(P)H dehydrogenase (quinone)”, “antigen processing and presentation” and other processes. Thus, immune system involvement, leukocyte activation, and potential immune dysfunction pathways were enriched in CRAO blood samples. KEGG analysis of both datasets also revealed transcriptional regulatory pathways, including “transcriptional misregulation in cancer”, “viral life cycle”, and “type I diabetes mellitus”, as well as “VEGF signalling pathway”, "yersinia infection" and other immune/inflammatory pathways. The V–C group exhibited enrichment of additional immune/inflammatory pathways, such as “Toll-like receptor signalling” and “Th17 cell differentiation”. GSEA of DEGs revealed enrichment of pathways involved in chromatin segregation, including CENP-A-related pathways and “chromosome, centromeric core domain”. Furthermore, GSEA revealed enrichment of multiple MHC-related genes and antigen-presenting activity pathways in the V–C group, indicating that inflammatory responses play a role in CRAO (Fig. 2e–g). In summary, both groups demonstrated ribosomal pathway enrichment, and the V–C group uniquely displayed enrichment in antigen presentation and inflammatory pathways.

### Protein‒protein interaction (PPI) network analysis and hub gene filtration

**Figure 3:**
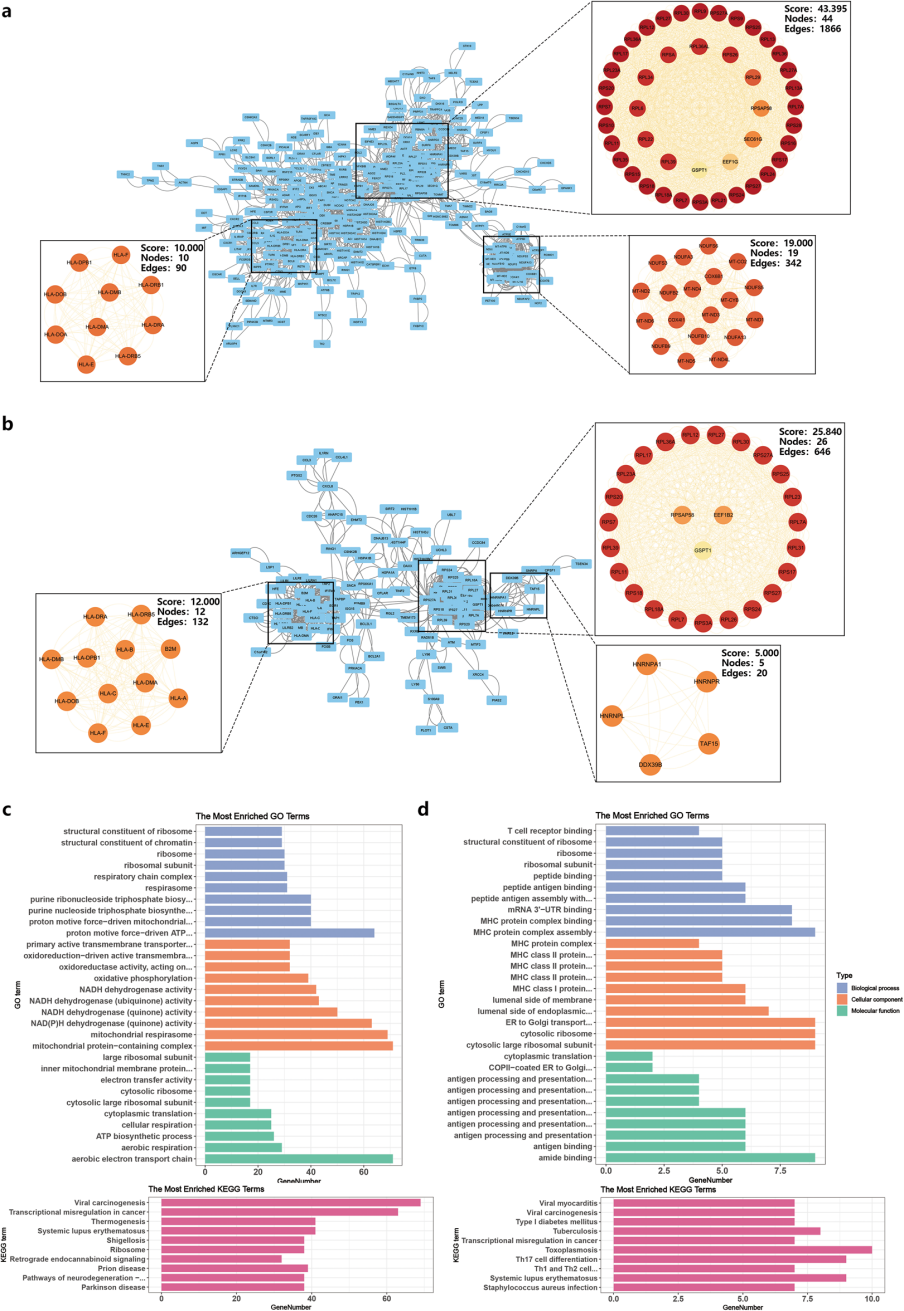
The PPI network. (**a**) The PPI network of common DEGs, and top three gene clustering modules in MCODE analysis of A–V group. (**b**) The PPI network of common DEGs, and top three gene clustering modules in MCODE analysis of V–C group. (**c** and **d**) GO and KEGG enrichment analysis of total modular genes.

To investigate PPIs among proteins encoded by the DEGs in CRAO, we constructed a PPI network using STRING (interaction score > 0.7) and visualized it in Cytoscape (the top 2,000 DEGs ranked by |log2-fold change| were analysed in the A–V group due to the large number of genes). Significant gene clustering modules were identified using the MCODE plug-ins (Fig. 3a and b). Enrichment analysis of the A–V group modules revealed their involvement in antigenic peptide synthesis, transcriptional regulation, protein synthesis, and NADH dehydrogenase activity (Fig. 3c). The V–C group exhibited a similar enrichment pattern, with additional emphasis on antigen recognition/presentation and MHC-associated responses (Fig. 3d). These findings indicate that ribosome-related genes and inflammatory responses may significantly contribute to CRAO development and progression.

### Selection and analysis of hub genes

**Figure 4:**
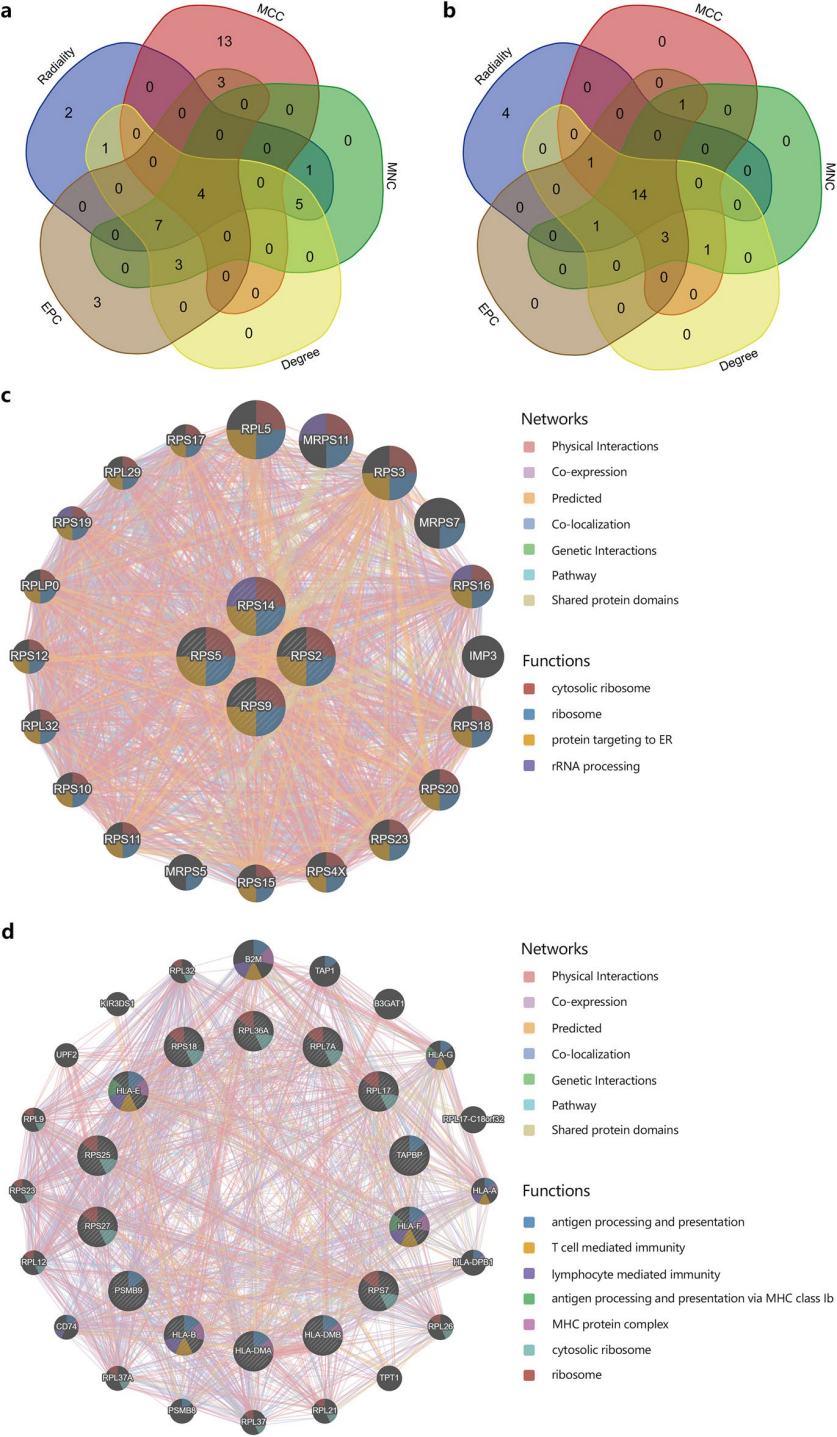
(**a**) The Venn diagram identified 3 candidates for hub genes in A–V group by five algorithms. (**b**) The Venn diagram identified 3 candidates for hub genes in V–C group by five algorithms. (**c** and** d**) The co-expression genes were analyzed via GeneMANIA. The predicted genes are located in the outer circle, and hub gene is in the inner circle.

To identify hub genes in CRAO, we used the CytoHubba plug-in and diverse algorithms to determine hub genes from the common DEGs (Tables 2 and 3). The notable hub genes of the A–V group were *RPS2, RPS5, RPS9*, and *RPS14* (Fig. 4a). The hub genes of the V–C group were *HLA-F, RPS25, RPS18, HLA-DMA, PSMB9, RPL36A, HLA-B, RPS7, RPL7A, HLA-E, TAPBP, RPS27, HLA-DMB, and RPL17* (Fig. 4b). We then analysed the coexpression network and related functions of these hub genes based on the GeneMANIA database, which revealed the top 20 most interacting genes. In the A–V group, the hub genes primarily functioned in various protein synthesis processes, including cytosolic ribosomes, ribosomes, protein targeting to the ER, and rRNA processing (Fig. 4c). The V–C group hub genes exhibited diverse functions, including immune response (T-cell-mediated immunity, antigen processing/presentation via MHC class Ib) and regulatory roles in MHC protein complexes, cytosolic ribosomes, ribosomes, and gene transcription/protein synthesis. These genes actively regulated cellular processes linked to gene expression, protein synthesis, and the immune response (Fig. 4d).

**Table 2:** The top 20 hub genes rank of A–V group in CytoHubba.

Radiality	MCC	MNC	Degree	EPC
RPS27A	RPL29	RPS27A	RPS27A	RPS27A
MYC	RPL13	RPS2	RPS2	RPS2
RPS14	RPL21	RPS3	RPS3	RPS14
RPS5	RPL7	RPS5	RPS5	RPS28
RPS18	RPL7A	RPS14	RPS14	RPS5
RPS3	RPL17	RPS18	RPS18	RPS3
RPS2	RPL27	RPS20	RPS20	RPS20
RPS20	RPL35A	RPS16	RPS16	RPS9
RPLP0	RPL34	RPS9	RPS9	RPS15
RPL23	RPS15A	RPL23	RPL23	RPL8
RPL11	RPS2	RPLP0	RPLP0	RPL12
RPS9	RPS3A	RPL11	RPL11	RPL35
RPL5	RPS9	RPL9	RPSA	RPL9
RPS16	RPS14	RPL8	RPL9	RPL17
H4C6	RPS24	RPL5	RPL5	RPL13A
RPSA	RPS10	RPSA	RPL8	RPL23A
H3C13	RPS19	RPL12	RPL12	RPS15A
EEF1A1	RPS28	RPL23A	RPL23A	RPL23
RPL23A	RPS5	RPL13A	RPL13A	RPS16
RPL13A	RPS17	H4C6	MYC	RPL6

**Table 3:** The top 20 hub genes rank of V–C group in CytoHubba.

Radiality	MCC	MNC	Degree	EPC
ISG15	RPS18	HLA-B	HLA-B	HLA-B
HLA-F	RPL7A	RPS18	ISG15	ISG15
PSMB9	RPS7	HLA-E	RPS18	HLA-E
TAP1	RPL7	HLA-F	HLA-E	HLA-F
IFITM1	RPL36A	HLA-DRA	HLA-F	PSMB9
HLA-B	RPS25	HLA-DMB	HLA-DRA	HLA-DMA
RPS18	RPS27	HLA-DMA	HLA-DMB	HLA-DMB
H4C6	RPL17	PSMB9	HLA-DMA	TAPBP
RPS7	RPL41	RPL7A	PSMB9	RPS18
RPL7A	GSPT1	RPS7	RPL7A	HLA-DRA
ABHD16A	HLA-B	RPL7	RPS7	RPS7
TAPBP	HLA-E	RPL36A	RPL7	RPL7A
HLA-E	HLA-F	RPS25	RPL36A	TAP1
HLA-DMA	HLA-DRA	RPS27	RPS25	RPL17
DDX39B	HLA-DMA	RPL17	RPS27	RPS27
HLA-DMB	HLA-DMB	TAP1	RPL17	RPS25
RPL36A	PSMB9	GSPT1	TAPBP	HLA-DPB1
RPS25	TAPBP	HLA-DPB1	RPL41	RPL7
RPS27	HLA-DPB1	TAPBP	TAP1	RPL41
RPL17	ISG15	RPL41	GSPT1	RPL36A

### Immune cell infiltration analysis

**Figure 5:**
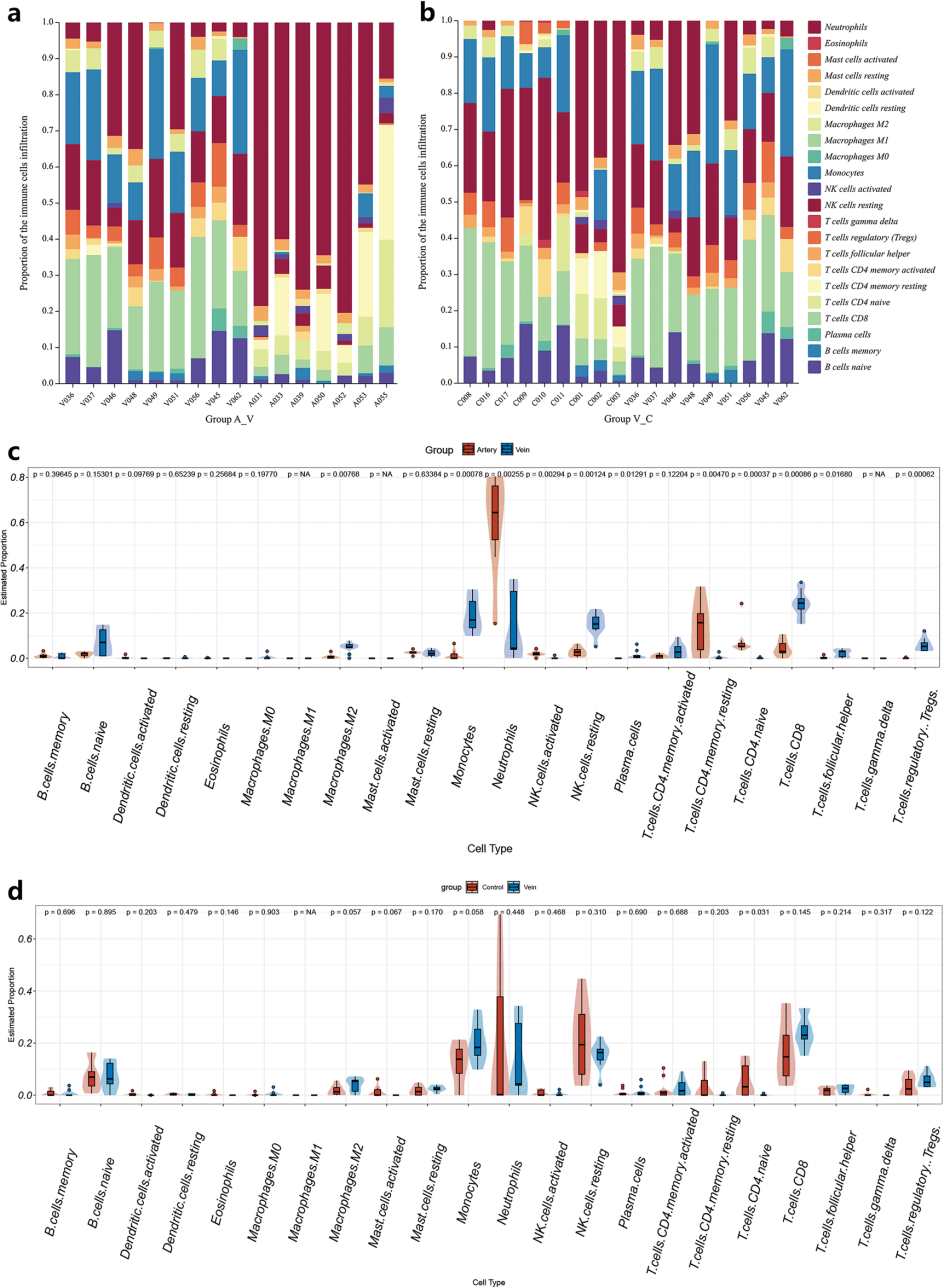
Composition of infiltrating immune cells in CRAO. (**a**) The proportion of immune cell populations in A–V group was determined by CIBERSORT. (**b**) The proportion of immune cell populations in V–C group was determined by CIBERSORT. (**c**) The proportion of immune cell populations in A–V group was determined by CIBERSORT. Red parts represent CRAO arterial blood samples. Blue parts represent CRAO venous blood samples. (**d**) The proportion of immune cell populations in V–C group was determined by CIBERSORT. Red parts represent cataract control blood samples. Blue parts represent CRAO venous blood samples.

To investigate the associations between CRAO and immune cell populations in peripheral blood, the proportions of 22 immune cell types in the ABS, VBS, and CON groups were quantified using CIBERSORT (Fig. 5a–d and Supplementary Figure S1). Compared to those in the VBS group, the numbers of neutrophils and CD4^+^ T memory cells were markedly increased in the ABS group (Fig. 5a and c). In addition, compared to that in the CON group, the number of naive CD4^+^ T cells was markedly increased in the VBS group (Fig. 5b and d). The GSEA results and gene information related to the antigen presentation pathway are shown in Supplementary Figure S2, Supplementary Table S1 and Supplementary Table S2.

## Discussion

The study of genes related to CRAO is crucial, yet limited research has hindered progress. Expanding research efforts using transcriptomic profiling is vital for identifying new biomarkers and therapeutic targets. For example, a recent RNA-seq study provided the first insights into retinal transcriptional changes in animal models of acute retinal artery ischaemia^27^. It identified potential therapeutic targets like *CXCL8* inhibition to mitigate retinal damage^27^. This study demonstrated the ability of transcriptomics to elucidate molecular mechanisms and develop treatment options for CRAO. Further transcriptomic research will enable better characterization of the complex CRAO gene expression landscape and improve diagnosis and management.

In this study, we identified 2691 DEGs in the A–V group and 146 DEGs in the V–C group by analysing two sets of genetic data. GO enrichment analysis revealed that the DEGs in both groups were enriched mainly in ribosomes and cytoplasmic ribosomes. The A–V group DEGs were associated with immune system activation, and the V–C group DEGs were associated with antigen presentation and binding. KEGG results suggested that these genes were primarily enriched in ribosomes and the Toll-like receptor signalling pathway. Interestingly, both the GO and KEGG pathway analyses suggested that ribosome-related genes may be involved in the pathogenesis of CRAO.

Hub gene and coexpression network analyses revealed multiple ribosomal proteins, including *RPS2*, *RPS5*, *RPS7*, *RPS9*, *RPS14*, *RPS18*, *RPS27*, *RPL7A*, *RPL17*, *and RPL36A*, as hub proteins. These proteins regulate the cell cycle, differentiation, apoptosis, and development^28–31^. *RPS7*^32^, *RPS9*^33^, and *RPS27*^34–36^ specifically participate in the *p53* and *NF-κB* pathways, which are important for cellular stress and inflammation. Ischaemic conditions upregulate various ribosomal proteins in the central nervous system, such as *RPS1*, *RPS3*, *RPS6*, and *RPS9*. *RPS3*^37,38^ and *RPS6*^39^ demonstrate neuroprotective effects. This ribosomal protein upregulation provides insights into the enrichment of ribosomal pathways in CRAO. Similarly, in myocardial infarction, the death of resident macrophages induces neutrophil and monocyte-derived macrophage repopulation of the infarct area^40^, causing inflammation, cytokine release, and post-MI cell death^41^. However, as tissue repair is initiated, macrophages switch from proinflammatory to pro-healing phenotypes, promoting collagen deposition, matrix remodelling, and scar formation^42,43^. This macrophage phenotype change may explain the observed ribosomal protein enrichment in CRAO.

The upregulation of ribosomal proteins observed in CRAO likely represents an endogenous protective response, as ribosomal proteins play important cytoprotective roles in other ischaemic conditions. Ribosomal protein *S6* phosphorylation impacts neuronal survival and axon regeneration in cerebral ischaemia‒reperfusion injury^44^. As metal binding proteins, the ribosomal proteins *S6*, *S3a*, and *S4X* regulate ribosomal RNA stability and are linked to cellular oxidative stress and inflammation^45^. Additionally, exogenous ribosomal proteins can alleviate ischaemia/hypoxia-induced cell damage. A peptide from ribosomal L23a inhibits myocardial apoptosis and ischaemia‒reperfusion injury^46^. Ribosomal *L26* binds *p53* mRNA to promote *p53* translation in hypoxia-induced apoptosis^46^. In summary, ribosomal proteins mediate cell stress responses, growth regulation, and cell survival in ischaemic diseases. Thus, modulating their function may mitigate ischaemia‒reperfusion injury.

The major histocompatibility complex (MHC) comprises highly polymorphic gene clusters encoding proteins involved in intercellular recognition and antigen presentation and is called the HLA complex in humans. Direct allorecognition involves recipient T cells recognizing alloantigens presented by endothelial cell HLA molecules^47^. While most studies have focused on immune rejection post transplantation, little research has been conducted on ischaemic diseases^48,49^. Recent evidence indicates that in patients with ischaemic stroke, CD8^+^ and CD4^+^ T cells are activated in the cerebrospinal fluid, and T-cell migration and infiltration are guided by HLA molecule interactions^50^. *PSMB9,* located in the MHC class II region, regulates apoptosis and MHC class I antigen presentation by encoding the immunoproteasome catalytic subunit *LMP2. LMP2* attenuates ischaemia/hypoxia-induced blood‒brain barrier injury through *Wnt/β-catenin* signalling activation^51^. Transporter associated with antigen processing (*TAP*) binding protein (*TAPBP*) encodes tapasin, a key MHC-I pathway component^52,53^, that mediates inflammatory injury via CD8^+^ T-cell infiltration^52,54^. A novel partial MHC II structure, *DRmQ*, promotes ischaemic stroke neuroprotection by inhibiting neuroantigen-specific T cells and blocking cytotoxic monocytes/macrophages^55^. *DRmQ* can also reduce inflammation and infarct volume in ischaemia by inhibiting T-cell activation and cytokine secretion^56^. These findings underscore the roles of MHC-related genes such as *PSMB9* and *TAPBP* in regulating the early inflammatory response of CRAO through MHC-mediated T-cell activation.

Our studies revealed an imbalance between neutrophils and CD4^+^ T cells in patients with CRAO, suggesting that inflammatory responses and immune cell infiltration may contribute to CRAO progression^57,58^. Research has shown that CD4^+^ T cells are activated postmyocardial infarction to promote myocardial wound healing^59^. Additionally, CD4^+^ T cells can delay the ischaemic infiltration of B cells and alleviate poststroke cognitive decline^60^, potentially playing an important neuroprotective role. In summary, our findings imply that immune activation participates in the pathogenesis of CRAO. CD4^+^ T cells may promote inflammatory responses in the early stages of CRAO but also mediate repair and protection in later stages.

The present study, however, has several limitations. First, due to limitations in collecting clinical samples, retinal tissue samples from patients with CRAO were not available, and PBMCs were used as a substitute. Therefore, future studies should aim to obtain patient cooperation and consent. Second, although key pathways, hub molecules, and immune cells identified in this study were identified in human specimens, additional laboratory studies, particularly animal experiments, are necessary to confirm their pathogenic significance. Furthermore, large-scale clinical trials are needed for further investigation of the underlying mechanisms involved. Finally, this study could not determine whether the apoptosis pathway induces immune cell infiltration or confirm the involvement of immune cells in the apoptotic process. Further research is warranted to elucidate the potential mechanisms involved.

## Conclusions

This transcriptomics study identified ribosomal proteins, HLA genes, and antigen processing molecules as potential biomarkers for CRAO screening and prognosis. Their dysregulation can collectively result in retinal cell dysfunction, immune-mediated damage, and CRAO progression. Specifically, decreased ribosomal protein synthesis may impact ischaemic retinal injury through insufficient production of proteins required to maintain retinal cell function^61,62^. Aberrant MHC gene expression can also dysregulate antigen presentation, activate proinflammatory T cells^50,63^, and exacerbate ischaemia‒reperfusion damage. Therefore, normalizing the expression of ribosomal proteins and MHC genes may mitigate retinal damage in patients with CRAO by attenuating cytotoxic inflammation. In summary, these transcriptomic signatures provide clinically relevant insights into CRAO mechanisms and offer biomarker-guided avenues for personalized diagnostic and therapeutic strategies. Validating the functional significance and clinical utility of these biomarkers will be key to translating these findings into precision medicine approaches for improved CRAO management.

### Supplementary Information

Supplementary Information.

## Data Availability

The datasets generated during and analysed during the current study are available in the Gene Expression Omnibus (GEO) repository, https://www.ncbi.nlm.nih.gov/geo/query/acc.cgi?acc=GSE247506.
