# Supplementary material for: Peripheral blood transcriptomic analysis identifies potential inflammation and immune signatures for central retinal artery occlusion
Source: Sci Rep. 2024 Mar 28;14:7398. doi: 10.1038/s41598-024-57052-8 (PMC10978867; doi:10.1038/s41598-024-57052-8)
Supplement: Supplementary file 1 — Supplementary Information. [file 41598_2024_57052_MOESM1_ESM.pdf]

# Peripheral Blood Transcriptomic Analysis Identifies Potential Inflammation and Immune Signatures for Central Retinal Artery Occlusion

*Jiaqing Feng*[1†], *Ying Li*[1†], *Chuansen Wang*[1], *Yuedan Wang*[1], *Yuwei Wan*[1], *Mengxue Zheng*[1], *Ting Chen*[1*], *Xuan Xiao*[12*]

1 Department of Ophthalmology, Renmin Hospital of Wuhan University, Wuhan, Hubei, China.

2 Department of Clinical Laboratory, Institute of Translational Medicine, Renmin Hospital of Wuhan University, Wuhan, China.

* **Correspondence:** Xuan Xiao, Department of Ophthalmology, Renmin Hospital of Wuhan University, No. 238 Jie Fang Road, Wuhan 430060, Hubei, China. Tel: (86) 027-88041911, Email: xiaoxuan1111@whu.edu.cn; Ting Chen, Department of Ophthalmology, Renmin Hospital of Wuhan University, No. 238 Jie Fang Road, Wuhan 430060, Hubei, China. Email: ct19870629@hotmail.com.

† These authors contributed equally.

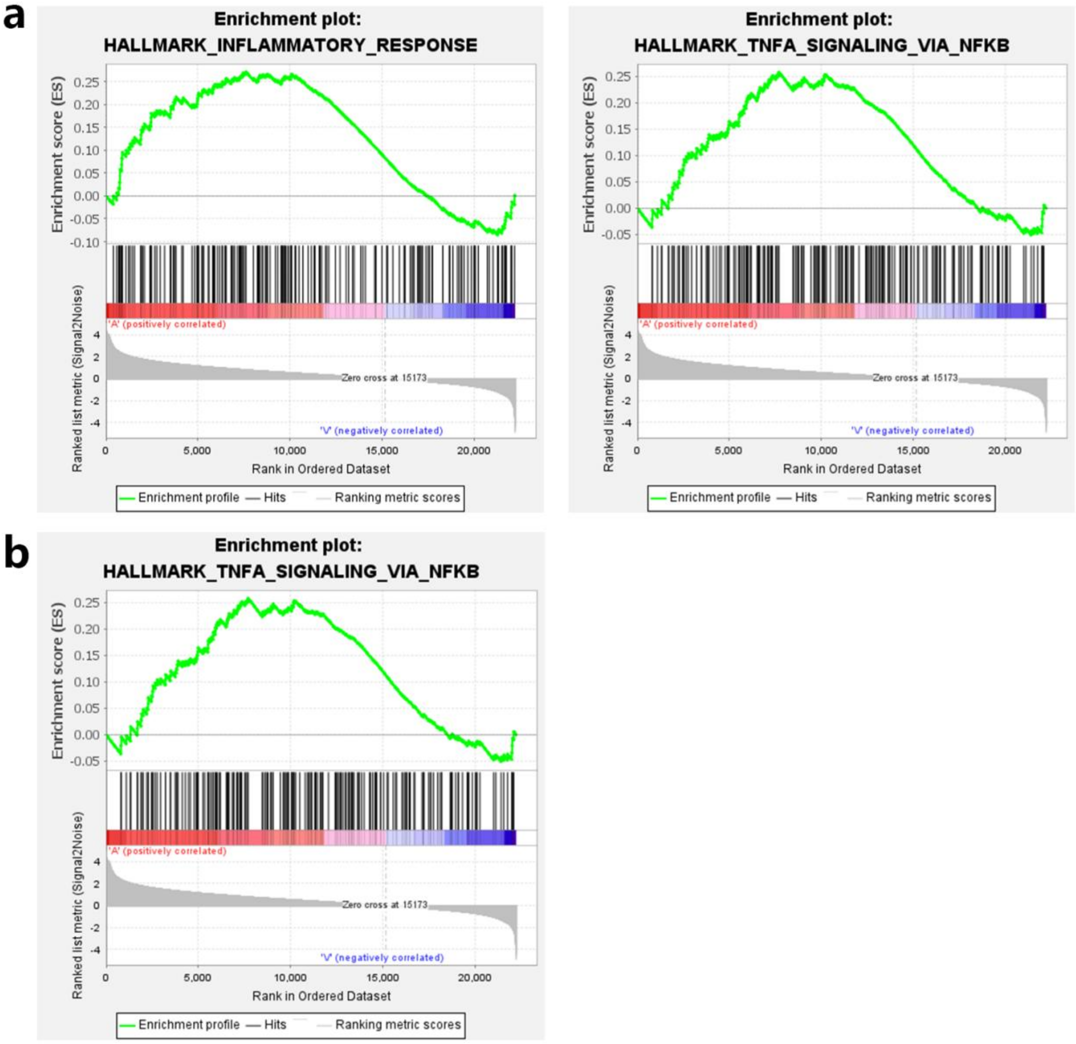


**Supplementary Figure S1** **a** GSEA results indicate activation of the antigen-presenting activity pathway in A-V group (INFLAMMATORY_RESPONSE: *NES* = 0.93 *p-value* = 0.6966967 *FDR q-value* = 1.0; TNFA_SIGNALING_VIA_NFKB: *NES* = 0.89 *p-value* = 0.76984924 *FDR q-value* = 1.0). **b** GSEA results indicate activation of the antigen-presenting activity pathway in V-C group (TNFA_SIGNALING_VIA_NFKB: *NES* = NaN *p-value* = NaN *FDR q-value* = 1.0).

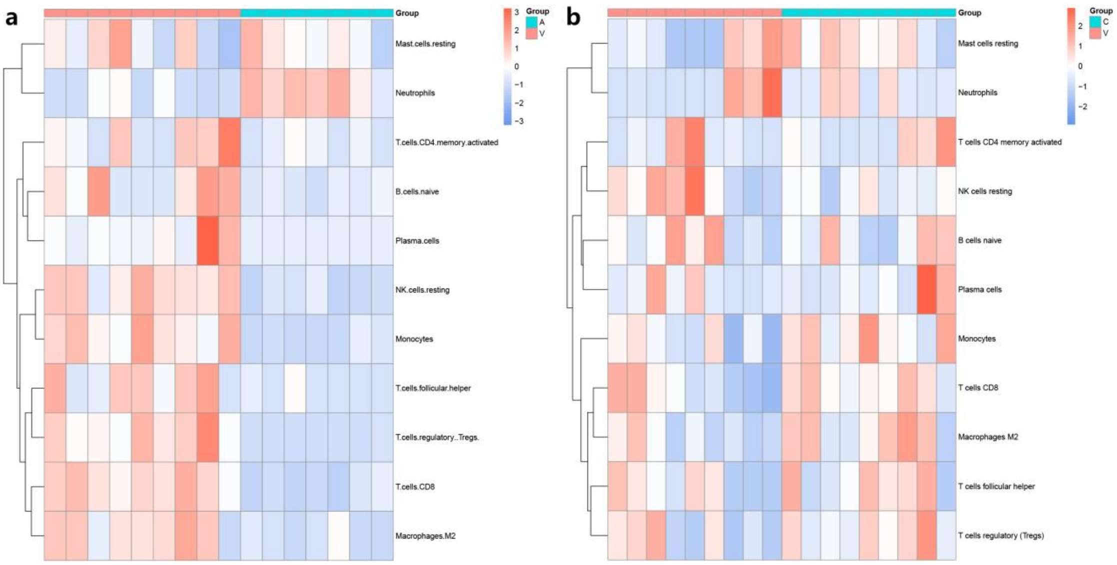


**Supplementary Figure S2** Heatmap of CRAO infiltrating immune cell composition. **a** The heatmap of immune cell populations in A_V group. Red parts represent CRAO venous blood samples. Blue parts represent CRAO arterial blood samples. **b** The heatmap of immune cell populations in V_C group. Red parts represent CRAO venous blood samples. Blue parts represent cataract blood samples.

**Supplementary Table S1** Results of GSEA core enrichment gene and its differential expression for A-V group.

| **GSEA Results Summary** | | | | | **Differential gene expression results** | |
|---|---|---|---|---|---|---|
| SYMBOL | RANK IN GENE LIST | RANK METRIC SCORE | RUNNING ES | CORE ENRICHMENT | logFC | adj.P.Val |
| ABCA1 | 1097 | 2.147211075 | 0.09906394 | Yes | 3.141635106 | 2.68E-06 |
| ABI1 | 7433 | 0.820745647 | 0.26445752 | Yes | 0.541221199 | 0.119620065 |
| ACVR1B | 2392 | 1.646935701 | 0.15405758 | Yes | 0.965907307 | 0.001425114 |
| AHR | 5011 | 1.141373754 | 0.21982124 | Yes | 1.383316598 | 0.000314065 |
| AQP9 | 3528 | 1.38896811 | 0.19556804 | Yes | 3.617131494 | 0.000192926 |
| ATP2A2 | 5926 | 1.014550924 | 0.2438633 | Yes | 0.856729591 | 0.006302511 |
| ATP2B1 | 2765 | 1.555349827 | 0.18636551 | Yes | 2.600997436 | 1.24E-06 |
| ATP2C1 | 2960 | 1.505529642 | 0.1867694 | Yes | 1.312046025 | 6.96E-06 |
| BTG2 | 5611 | 1.055390477 | 0.23295575 | Yes | 1.811204001 | 0.001419239 |
| CCRL2 | 7297 | 0.837295175 | 0.2604508 | Yes | 0.386003875 | 0.412323814 |
| CD55 | 5225 | 1.108021736 | 0.22392014 | Yes | 1.467803846 | 0.002471534 |
| CLEC5A | 7546 | 0.804528773 | 0.2693335 | Yes | 0.088430265 | 0.808936001 |
| CSF1 | 1324 | 2.024342775 | 0.11384494 | Yes | 0.847013883 | 0.041268692 |
| CSF3R | 5243 | 1.105030298 | 0.22989944 | Yes | 3.153644678 | 1.03E-05 |
| CXCR6 | 6189 | 0.978307962 | 0.2440971 | Yes | 0.905501116 | 0.028739558 |

| | | | | | | |
|---|---|---|---|---|---|---|
| CYBB | 7448 | 0.817315936 | 0.26881537 | Yes | 1.496759154 | 0.006128574 |
| EIF2AK2 | 6546 | 0.933136344 | 0.25705937 | Yes | 2.864312087 | 2.02E-05 |
| FPR1 | 4954 | 1.149717212 | 0.20130765 | Yes | 3.516095738 | 0.000257031 |
| GNAI3 | 1553 | 1.925397038 | 0.12737393 | Yes | 1.803412017 | 3.96E-05 |
| GPR132 | 2472 | 1.626081467 | 0.18041456 | Yes | 0.843912468 | 0.009580302 |
| HIF1A | 3524 | 1.390106559 | 0.1872199 | Yes | 1.775625921 | 0.000206483 |
| IFNAR1 | 5056 | 1.135987878 | 0.22476599 | Yes | 0.627644725 | 0.057636602 |
| IL10RA | 7614 | 0.795042813 | 0.2711533 | Yes | 1.307035768 | 0.01207924 |
| IL1B | 3507 | 1.392635822 | 0.17945397 | Yes | 3.522022455 | 0.000409548 |
| IL1R1 | 6812 | 0.898425043 | 0.25053716 | Yes | 2.152171574 | 0.000663421 |
| IL7R | 5815 | 1.027552605 | 0.23636295 | Yes | 2.947100089 | 0.000541439 |
| IRAK2 | 5566 | 1.06027174 | 0.22850332 | Yes | 0.295853655 | 0.37051445 |
| IRF1 | 2007 | 1.765646815 | 0.15075237 | Yes | 3.164466186 | 2.40E-07 |
| ITGA5 | 769 | 2.401807785 | 0.043723777 | Yes | 1.731899072 | 2.64E-06 |
| KCNA3 | 4688 | 1.191090584 | 0.1991129 | Yes | 2.240521964 | 5.33E-05 |
| KCNJ2 | 4196 | 1.270138383 | 0.21408992 | Yes | 4.329387383 | 7.29E-07 |
| KIF1B | 896 | 2.287798405 | 0.09501246 | Yes | 2.067956983 | 1.13E-05 |
| KLF6 | 3808 | 1.335377336 | 0.2160146 | Yes | 1.888570742 | 2.72E-05 |
| LCP2 | 696 | 2.475392818 | 0.017488128 | Yes | 3.575744439 | 1.24E-07 |
| LDLR | 6517 | 0.937031865 | 0.25262856 | Yes | 0.743938087 | 0.063418353 |
| LPAR1 | 5869 | 1.022102833 | 0.24020417 | Yes | 0.553068201 | 0.081759108 |
| LYN | 3664 | 1.361420751 | 0.19776559 | Yes | 2.067738137 | 0.001066423 |
| MEFV | 2096 | 1.735894918 | 0.15736735 | Yes | 2.488775707 | 0.000354728 |
| MET | 6333 | 0.959333599 | 0.24939145 | Yes | 0.529685712 | 0.215330595 |
| MXD1 | 3749 | 1.34374249 | 0.21048643 | Yes | 2.488940532 | 0.000469561 |
| NAMPT | 4972 | 1.147269487 | 0.20754497 | Yes | 3.773522148 | 4.96E-05 |
| NFKB1 | 2462 | 1.628083944 | 0.17089002 | Yes | 1.521193005 | 8.83E-06 |
| NOD2 | 866 | 2.310437441 | 0.08235242 | Yes | 2.829085731 | 2.55E-06 |
| P2RY2 | 6067 | 0.996492803 | 0.24360518 | Yes | 0.92908312 | 0.024795518 |
| PDE4B | 4133 | 1.278743625 | 0.20914154 | Yes | 2.637240348 | 3.31E-05 |
| PIK3R5 | 1910 | 1.799359918 | 0.13331304 | Yes | 2.373165674 | 0.000173209 |
| PLAUR | 7155 | 0.852117896 | 0.25653565 | Yes | 0.927257083 | 0.031541056 |
| PROK2 | 6321 | 0.961016893 | 0.24403018 | Yes | 2.651658457 | 0.013664541 |
| PSEN1 | 748 | 2.420135021 | 0.029959455 | Yes | 2.135943982 | 1.82E-08 |
| PTAFR | 3689 | 1.354669571 | 0.2049525 | Yes | 1.715867881 | 0.002601961 |
| PTGER4 | 1887 | 1.806926012 | 0.12331924 | Yes | 2.491776196 | 1.60E-06 |
| PTPRE | 1923 | 1.794283271 | 0.14372905 | Yes | 2.367904076 | 3.72E-06 |
| RAF1 | 1236 | 2.064934254 | 0.105422735 | Yes | 2.845213385 | 3.96E-06 |
| RASGRP1 | 4996 | 1.142953396 | 0.21348399 | Yes | 1.870479611 | 9.47E-05 |
| RNF144B | 3194 | 1.453426838 | 0.18508752 | Yes | 1.62873792 | 0.007993741 |
| ROS1 | 406 | 2.951932192 | -3.69E-04 | Yes | 1.932636577 | 2.29E-09 |
| SELL | 1441 | 1.973749518 | 0.12064381 | Yes | 3.278387231 | 1.08E-06 |
| SEMA4D | 595 | 2.584956169 | 0.006899986 | Yes | 3.06180303 | 1.47E-08 |

| | | | | | | |
|---|---|---|---|---|---|---|
| SERPINE1 | 2656 | 1.578018546 | 0.18175974 | Yes | 0.720471784 | 0.051132927 |
| SGMS2 | 862 | 2.313435316 | 0.06837576 | Yes | 2.124655617 | 6.72E-06 |
| SLC11A2 | 7248 | 0.842422962 | 0.25751182 | Yes | 0.650555433 | 0.05861646 |
| SLC1A2 | 6853 | 0.892670512 | 0.25417694 | Yes | -0.029292168 | 0.668085237 |
| SLC31A1 | 5327 | 1.092867494 | 0.23281328 | Yes | 0.421075682 | 0.205208339 |
| SLC31A2 | 2451 | 1.632159829 | 0.16139854 | Yes | 5.291990898 | 3.13E-07 |
| SLC7A1 | 4828 | 1.168378711 | 0.19995004 | Yes | 0.585112055 | 0.004330867 |
| TLR1 | 772 | 2.397631168 | 0.05827836 | Yes | 5.334255732 | 8.72E-06 |
| TLR2 | 6938 | 0.880274236 | 0.2557469 | Yes | 1.933818351 | 0.006435412 |
| TNFAIP6 | 5806 | 1.029018402 | 0.230449 | Yes | 2.469566449 | 0.001656803 |
| TNFRSF9 | 7360 | 0.829441488 | 0.26270732 | Yes | 0.387166147 | 0.436791585 |
| TNFSF10 | 6462 | 0.943301022 | 0.24935229 | Yes | 1.838071234 | 0.000557647 |
| TNFSF15 | 6997 | 0.872230768 | 0.25844607 | Yes | 0.019777787 | 0.891903742 |

**Supplementary Table S2** Results of GSEA core enrichment gene and its differential expression for V_C group.

| **GSEA Results Summary** | | | | | **Differential gene expression results** | |
|---|---|---|---|---|---|---|
| SYMBOL | RANK IN GENE LIST | RANK METRIC SCORE | RUNNING ES | CORE ENRICHMENT | logFC | adj.P.Val |
| BCL2A1 | 107 | 0.716404855 | 0.06213767 | Yes | 0.578938882 | 0.391172768 |
| BTG2 | 2957 | 0.166483715 | 0.20167959 | Yes | 1.811204001 | 0.001419239 |
| CCL2 | 1549 | 0.321524501 | 0.18255685 | Yes | 0.363144308 | 0.342121264 |
| CCL4 | 997 | 0.402795255 | 0.15073267 | Yes | -2.275404287 | 0.010466359 |
| CCNL1 | 1757 | 0.295993149 | 0.18899953 | Yes | 1.00931309 | 0.03816077 |
| CD69 | 2312 | 0.231726542 | 0.1989278 | Yes | -1.4044371 | 0.124069402 |
| CDKN1A | 2665 | 0.193001255 | 0.19005321 | Yes | -1.198155723 | 0.023679996 |
| CEBPB | 855 | 0.426316679 | 0.1286406 | Yes | 0.980862988 | 0.116714322 |
| CEBPD | 1218 | 0.366579384 | 0.16748759 | Yes | 1.541821163 | 0.018314138 |
| CXCL1 | 1924 | 0.273937672 | 0.19100183 | Yes | -1.284998311 | 0.078609026 |
| CXCL10 | 2450 | 0.217732832 | 0.1964485 | Yes | 0.461947852 | 0.452263854 |
| DRAM1 | 2829 | 0.177917361 | 0.19863418 | Yes | 0.074662659 | 0.868253801 |
| DUSP1 | 1283 | 0.358453602 | 0.18326537 | Yes | 3.783114729 | 0.000672007 |
| DUSP2 | 2249 | 0.238595039 | 0.19363146 | Yes | -1.164016923 | 0.115783919 |
| EGR1 | 461 | 0.523854077 | 0.09657043 | Yes | -1.306069513 | 0.153606818 |
| EGR3 | 1962 | 0.268510669 | 0.19392818 | Yes | 0.044739957 | 0.961744227 |
| EIF1 | 501 | 0.51113534 | 0.11246063 | Yes | -1.903461711 | 3.28E-06 |
| FOS | 59 | 0.77938664 | 0.03945097 | Yes | 3.397149896 | 0.000890374 |
| FOSB | 483 | 0.516041696 | 0.1044669 | Yes | -0.462882977 | 0.551729137 |
| G0S2 | 309 | 0.575639606 | 0.08502607 | Yes | -1.33623658 | 0.2474329 |
| GADD45B | 1659 | 0.306854457 | 0.1883229 | Yes | -0.870288289 | 0.000829997 |

| | | | | | | |
|---|---|---|---|---|---|---|
| GCH1 | 1112 | 0.383460909 | 0.16596338 | Yes | -0.590784739 | 0.198180266 |
| ID2 | 1434 | 0.335350037 | 0.18221335 | Yes | -0.809343581 | 0.100217421 |
| IER2 | 223 | 0.621659636 | 0.07901002 | Yes | -0.561776464 | 0.334800777 |
| IER5 | 1225 | 0.365484148 | 0.17348261 | Yes | -0.932675526 | 0.02757171 |
| IFIH1 | 2251 | 0.23846972 | 0.19767526 | Yes | 0.446416378 | 0.361688433 |
| IFNGR2 | 27 | 0.851051807 | 0.013368938 | Yes | -3.318928004 | 1.96E-07 |
| IL12B | 2729 | 0.187617376 | 0.19369216 | Yes | -0.037844729 | 0.574881622 |
| JUNB | 1015 | 0.400258034 | 0.16374154 | Yes | 1.323322949 | 0.026712472 |
| KLF10 | 160 | 0.663789332 | 0.0711618 | Yes | -1.775567065 | 0.002568476 |
| KLF2 | 388 | 0.546087801 | 0.09085283 | Yes | 0.680732544 | 0.046660175 |
| KLF4 | 517 | 0.504838049 | 0.12043707 | Yes | 0.006525316 | 0.993095228 |
| KLF9 | 2719 | 0.188567623 | 0.19088314 | Yes | -0.308671976 | 0.518781218 |
| MAFF | 2911 | 0.170718446 | 0.20086554 | Yes | -1.398993 | 0.003424055 |
| MCL1 | 773 | 0.443686187 | 0.12500365 | Yes | 1.437451557 | 0.014085754 |
| MYC | 1011 | 0.40070495 | 0.15701419 | Yes | -2.061210597 | 0.000642111 |
| NFKBIA | 888 | 0.420115471 | 0.1417233 | Yes | 0.005267876 | 0.994942397 |
| NINJ1 | 863 | 0.424822509 | 0.13560778 | Yes | 0.023436021 | 0.968698606 |
| PER1 | 2786 | 0.181641296 | 0.19748802 | Yes | -1.245185127 | 0.005763477 |
| PPP1R15A | 980 | 0.4055897 | 0.14455134 | Yes | 0.315801107 | 0.497434683 |
| PTGS2 | 1269 | 0.360155642 | 0.17770834 | Yes | 1.881849355 | 0.029348992 |
| SAT1 | 98 | 0.728286326 | 0.05021595 | Yes | 0.954573846 | 0.09415075 |
| SOD2 | 1882 | 0.279203385 | 0.18816383 | Yes | 2.282687003 | 0.004032491 |
| STAT5A | 2905 | 0.170983478 | 0.1981649 | Yes | 0.113527278 | 0.608535951 |
| TAP1 | 43 | 0.820997536 | 0.026766714 | Yes | -5.917470977 | 8.81E-09 |
| TNIP1 | 2755 | 0.185181916 | 0.19573382 | Yes | 0.378573441 | 0.151179659 |
| TNIP2 | 2230 | 0.240052119 | 0.19035645 | Yes | -0.616870297 | 0.026563804 |
| TRIB1 | 2081 | 0.255135477 | 0.19295187 | Yes | 1.621826908 | 0.002192546 |
| YRDC | 1605 | 0.31503278 | 0.18546464 | Yes | -0.502555785 | 0.092915013 |
| ZFP36 | 558 | 0.49437356 | 0.12710035 | Yes | 1.049881855 | 0.144460695 |
